# Pooled-Analysis of Association of Sievers Bicuspid Aortic Valve Morphology With New Permanent Pacemaker and Conduction Abnormalities After Transcatheter Aortic Valve Replacement

**DOI:** 10.3389/fcvm.2022.884911

**Published:** 2022-05-26

**Authors:** Jiajun Zhang, Xiaoxing Li, Feng Xu, Yuguo Chen, Chuanbao Li

**Affiliations:** ^1^Department of Emergency Medicine and Chest Pain Center, Cheeloo College of Medicine, Qilu Hospital of Shandong University, Jinan, China; ^2^Key Laboratory of Emergency and Critical Care Medicine of Shandong Province, Qilu Hospital of Shandong University, Jinan, China; ^3^Key Laboratory of Cardiovascular Remodeling and Function Research, Qilu Hospital of Shandong University, Jinan, China; ^4^Department of Geriatrics, Qilu Hospital of Shandong University, Jinan, China

**Keywords:** bicuspid aortic valve, transcatheter aortic valve replacement, conduction abnormalities, aortic stenosis (AS), Sievers classification, pacemaker

## Abstract

**Background:**

Studies on the association of Sievers bicuspid aortic valve (BAV) morphology with conduction disorders after transcatheter aortic valve replacement (TAVR) have not reached consensus.

**Methods:**

We here performed a pooled-analysis to explore whether Sievers type 1 BAV morphology increased the risk of post-TAVR conduction abnormalities and permanent pacemaker implantation (PPI) compared to type 0. Systematic literature searches through EMBASE, Medline, and Cochrane databases were concluded on 1 December 2021. The primary endpoint was post-TAVR new PPI and pooled as risk ratios (RRs) and 95% confidence intervals (CIs). Conduction abnormalities as the secondary endpoint were the composites of post-TAVR PPI and/or new-onset high-degree of atrial-ventricle node block and left-bundle branch block. Studies that reported incidence of outcomes of interest in both type 1 and type 0 BAV morphology who underwent TAVR for aortic stenosis were included.

**Results:**

Finally, nine studies were included. Baseline characteristics were generally comparable, but type 1 population was older with a higher surgical risk score compared to type 0 BAV morphology. In the pooled-analysis type 1 BAV had significantly higher risk of post-TAVR new-onset conduction abnormalities (*RR* = 1.68, 95%*CI* 1.09–2.60, *p* = 0.0195) and new PPI (*RR* = 1.97, 95%*CI* 1.29–2.99, *p* = 0.0016) compared to type 0. Random-effects univariate meta-regression indicated that no significant association between baseline characteristics and PPI.

**Conclusion:**

Sievers type 1 BAV morphology was associated with increased risk of post-TAVR PPI and conduction abnormalities compared to type 0. Dedicated cohort is warranted to further validate our hypothesis.

## Introduction

Large randomized-controlled trials have proved the safety and efficacy of transcatheter aortic valve replacement (TAVR) for native tricuspid aortic valve stenosis (1, 2). Nevertheless, the bicuspid aortic valve (BAV) as the most common congenital cardiac anomaly was excluded from the pivotal trials (1–3). Clinical observations and meta-analyses have demonstrated that patients with BAV stenosis undergoing TAVR had comparable 30-day mortality to patients with tricuspid aortic valve stenosis (4). Observational registry studies evaluating the usefulness of TAVR for bicuspid aortic stenosis showed no statistical difference between surgical aortic valve replacement (SAVR) and TAVR in early mortality; however, the problem of post-procedural conduction abnormalities was tough and unsolved for TAVR (5–7).

Post-TAVR new-onset conduction disorders like high-degree atrial-ventricle node block (HAVB) and new permanent pacemaker implantation (PPI) were associated with increased adverse events (8). Procedure characteristics, such as lower implantation and oversizing of the implanted valve, are recognized risk factors for conduction abnormalities (9). Nevertheless, the association of Sievers BAV morphology with post-TAVR conduction disorders was controversial and poorly discovered. Ou et al. suggested type 1 BAV morphology as a strong predictor of HAVB (10). In contrast, one pooled-analysis demonstrated no significant difference in the incidence of post-TAVR new PPI between type 1 and type 0 BAV morphology but the primary endpoint of that study was 30-day mortality actually, which spoiled reliability of the conclusion (11). Accordingly, we performed a pooled-analysis focusing on post-TAVR conduction abnormalities and their association with Sievers BAV morphology.

## Method

The systematic review and pooled-analysis were conducted according to the Preferred Reporting Items for Systematic Reviews and Meta-Analyses (PRISMA) (12) and recommendations from Meta-Analyses of Observational Studies in Epidemiology (13).

### Search Strategy and Eligibility Criteria

Systematic literature searches through EMBASE, Medline, and Cochrane databases were concluded on December 1, 2021. The search strategy included (1) “transcatheter aortic valve implantation” OR “transcatheter aortic valve replacement” OR “percutaneous aortic” OR “transcatheter aortic valve,” (2) “conduction” OR “block” OR “pacemaker,” (3) “bicuspid,” then combined (1) AND (2) AND (3). A manual search was performed using references in published articles and conferences to identify potentially relevant research.

The search results were screened and viewed in the title and abstract first to identify relevant studies. Case reports and case serials were not qualified for screening. All identified relevant studies were then placed under full-text review to further validate the eligibility.

Based on Population, Intervention, Control, Outcome, and Study Design (PICOS), studies were eligible if they met the following criteria: (1) enrolling BAV population; (2) undergoing TAVR for aortic stenosis; (3) available incidence of post-TAVR PPI or conduction abnormalities in Sievers type 1 and type 0 BAV morphology.

### Outcomes of Interest

The primary outcome of interest was post-TAVR new PPI. The occurrence of high-degree atrial-ventricle node block (HAVB) or in some centers left-bundle branch block (LBBB) was an indication for PPI and might be associated with worse outcomes as well (8). We defined conduction abnormalities as the secondary endpoint composite of post-TAVR new PPI and/or new-onset HAVB and LBBB. To avoid repeat counting of HAVB and LBBB who subsequently received a permanent pacemaker, HAVB and LBBB were counted only in absence of reporting PPI or clearly stating the presented HAVB and LBBB were free from PPI. Definitions of outcomes were in line with the Valve Academic Research Consortium (VARC-2).

Specifically, type 1 BAV were further classified according to the location of fused raphe and cusp: L-R, raphe between left- and right-coronary cusp; R-N, raphe between right- and non-coronary cusp; L-N, raphe between left- and non-coronary cusp (14). The event rates across type 1 BAV subtypes were also collected and compared.

### Evaluating the Risk of Bias

Considering all included studies were observational, we used Newcastle-Ottawa Scale to assess the quality of included studies. Two authors (JZ and XL) independently completed databases searching and study screening and evaluations. Discrepancies were settled by a third researcher (YC).

**Figure 1:**
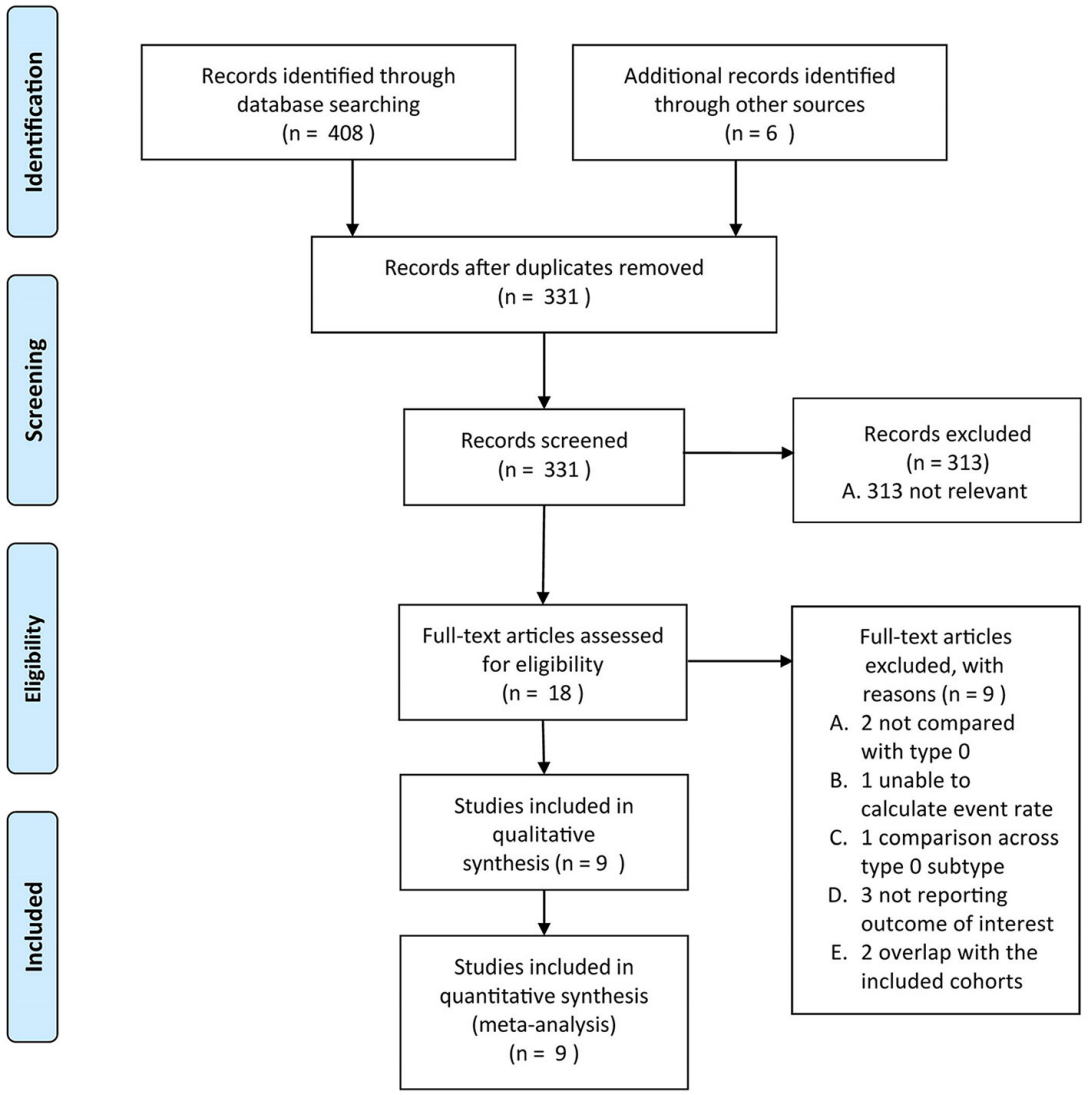
The Preferred Reporting Items for Systematic Reviews and Meta-Analyses (PRISMA) flowchart of the present study.

Duplicates and data overlap were confirmed by authors information and study start and end time, choices were made based on the evaluation of study quality, time period, and the number of subjects. The PRISMA flowchart is provided in Figure 1.

### Statistical Analysis

Following statistical analyses were completed on R (version 4.1.3) with Meta package (15). The pooled estimates of outcomes were expressed as risk ratios (RRs) and 95% confidence intervals (*CIs*) using the Mantel-Haenszel method with random-effects models. A two-tailed *p* <0.05 was considered statistically significant. Heterogeneity across studies was calculated by I^2^ and I^2^ > 50% was deemed unacceptable heterogeneity. Baujat plot and L'Abbé plot were drawn to visualize the origin of heterogeneity. Sensitivity analysis was conducted by removing one or more specific study/studies from the whole collection each time to evaluate the robustness of the pooled results and explore heterogeneity. As observational studies would introduce huge confounding effects and may lead to a biased estimate, we performed a random-effects univariate meta-regression to adjust for the influence of potential effect modifiers. Demographic characteristics and clinical parameters were selected as covariates. Subgroup analyses were performed according to valve type (self-expanded valve, SEV vs. balloon-expanded valve, BEV). The publication bias of each item was examined by funnel plots visually based on the symmetry.

## Results

After dedicated literature search and selection, 9 studies were included in the final pooled-analysis (9, 10, 16–22). Study quality was evaluated by Newcastle-Ottawa Scale (Table 1) and there was generally a low risk of bias in the included studies. Features of included studies were presented in Table 2.

**Table 1:** Quality assessment of eligible studies by Newcastle–Ottawa scale.

	**Jilaihawei et al**.	**Xiong et al**.	**Kumar et al**.	**Yoon et al**.	**Forrest et al**.	**Lelasi et al**.	**Hamdan et al**.	**Ou et al**.	**Guo et al**.
**Selection**									
Exposed cohort	1	1	1	1	1	1	1	1	1
Non-exposed	1	1	1	1	1	1	1	1	1
Exposure	1	1	0	1	1	1	1	1	1
Outcome	1	1	1	1	1	1	1	1	1
**Comparability**									
Most important factor	0	0	0	0	0	0	0	0	0
Additional factor	0	0	0	0	0	0	0	0	0
**Outcome**									
Assessment	1	0	0	1	1	0	0	1	1
Follow-up	1	1	1	1	1	1	1	1	1
Adequacy	1	1	1	1	1	1	1	1	1
**Sum**	7	6	5	7	7	6	6	6	7

**Table 2:** Overview of the included studies.

	**Jilaihawi et al**.	**Xiong et al**.	**Kumar et al**.	**Yoon et al**.	**Forrest et al**.	**lelasi et al**.	**Hamdan et al**.	**Ou et al**.	**Guo et al**.
No. of patients	130	80	67	1,034	150	243	67	181	209
Diagnosed by MDCT	70%	NA	NA	100%	100%	100%	100%	100%	100%
Type 0 morphology-no. (excluding prior PPI)*	21 (18)	46 (46)	11 (11)	107 (100)	14 (14)	25 (23)	17 (17)	102 (102)	99 (99)
Type 1 morphology-no. (excluding prior PPI)*	74 (60)	34 (34)	56 (56)	927 (866)	136 (132)	218 (198)	50 (50)	79 (79)	79 (79)
Type 1 morphology subtypes	NA	29 L-R; 3 N-R; 2N-L	NA	NA	107 L-R;27 N-R;2 N-L	NA	38 L-R; 12 N-R; 0 N-L	63 L-R; 16 Non-L-R	NA
Othermorphology-no^†^	25	0	0	NA	NA	0	0	0	31
**Patients enrollment**									
Time period	2005–2014	2012–2017	2017–2019	2016–2019	2018–2019	2013–2018	Since 2017	2015–2019	2016–2020
Data collection	Prospective	Retrospective	Retrospective	Prospective + retrospective	Prospective	Prospective	Prospective +retrospective	Prospective	Retrospective
Site	International; US, European, Asia, Canada	Single center in China	NA	International; European, Israel, US	Multicenter in US	Multicenter in European	Multi center in Israel	Single center in China	Multicenter in China
Exclusion criteria	NA	Prior PPI	NA	1) No pre-TAVR CT 2) Poor CT quality;	Predicted risk of 30-day mortality higher than 3.0	Type 2 and undetermined type	1) undetermined valve morphology; 2) priori PPI; 3) valve in valve; 4) no pre-TAVR CT	1) prior PPI 2) without pre- and post-TAVR CT	1) prior PPI; 2) transfer to open surgery; 3) poor quality of imaging; 4) valve other than SEV; 5) perioperative death

* *Number in the bracket means counts after excluding patients with prior PPI.*

† *Including type 2 and undetermined BAV morphology.*

*BAV, bicuspid aortic valve; L-R, left and right fusion; TAVR, transcatheter aortic valve replacement; MDCT, Multi-detector CT; PPI, permanent pacemaker implantation*.

### The Baseline and Procedural Characteristics Between Sievers Type 1 and Type 0

Generally, demographic characteristics were comparable between type 1 and type 0 BAV morphology; however, there was a relatively higher STS score in type 1 population. Patients across surgical risk profiles were included and most of them were in their 70's and had New York Heart Association functional class> 2. No patient included in the pooled-analysis had prior PPI. During the TAVR procedure pre-dilation was more frequently used in type 0 than type 1. No difference was found between type 1 and type 0 with regard to other procedural characteristics. In the 9 included studies, 4 were SEV-specific (9, 10, 16, 22) and in the other 5 studies both SEV and BEV were used. In the study of Yoon et al., statistically lower proportion of type 1 received SEV compared to type 0 (20), but in the other studies there was no such difference. The detailed characteristics could be found in Tables 3, 4.

**Table 3:** Baseline characteristics of patients with bicuspid aortic stenosis who underwent transcatheter aortic valve replace in the included studies.

	**Jilaihawi**		**Xiong**	**Kumar**	**Yoon**		**Forrest**		**Ielasi**		**Hamdan**	**Ou**	**Guo**		***MD* or RR†**	**95% *CI***	***p***
	**Type 0**	**Type 1**	Overall*	Overall*	**Type 0**	**Type 1**	**Type 0**	**Type 1**	**Type 0**	**Type 1**	Overall*	Overall*	**Type 0**	**Type 1**			
No.of pts	21	74	80	67	107	927	14	136	25	218	67	181	99	79	/	/	/
Age (yrs)	74.4 ± 7.3	76.1 ± 10.8	75 (70.0–77.0)	70.0 ± 9.9	69.5 ± 11.1	75.3 ± 8.9*	70.6 ± 4.1	70.3 ± 5.6	77.8 ± 9.3	79.1 ± 7.8	77.0 ± 8.8	73.1 ± 6.2	74.1 ± 7.0	76.3 ± 6.8	2.25	0.03–4.48	**0.0468**
Male *n*. (%)	11 ± 52.4	46 ± 62.2	47 ± 58.8	NA	63 ± 58.9	547 ± 59.0	5 ± 35.7	73 ± 53.7	19 ± 76.0	144 ± 66.1	42 ± 63	103 ± 56.9	54 ± 54.5	51 ± 64.6	1.03	0.91–1.18	0.611
STS PROMscore	4.2 (3.2–5.2)	5.1 (2.9–7.6)	7.7 ± 4.0	4.1 ± 3.7	3.0 ± 2.1	3.75 ± 3.4*	1.4 ± 0.5	1.4 ± 0.6	3.4 ± 1.8	4.5 ± 3.0	NA	6.3 ± 4.3	6.10 ± 3.8	7.77 ± 5.4	0.73	0.17–1.29	**0.0101**
NYHA III-IV no. (%)	18 (85.7)	60 (81.1)	NA	NA	72 (67.3)	667 (71.6)	2 (14.3)	39 (28.6)	17 (68.0)	146 (67.3)	NA	NA	NA	NA	1.03	0.92–1.14	0.614
Hypertension no. (%)	NA	NA	NA	NA	74 (69.2)	749 (80.8)	8 (57.1)	104 (76.5)	19 (76)	180 (72.6)	47 (70)	NA	49 (49.5)	36 (45.6)	1.13	1.02-1.25	**0.0221**
Diabetes no. (%)	8 (38.1)	15 (20.3)	NA	NA	32 (29.9)	232 (25.0)	5 (35.7)	32 (23.5)	6 (24)	45 (20.6)	20 (30)	NA	21 (21.2)	14 (17.7)	0.782	0.62–0.99	**0.0375**
Prior PCI no. (%)	4 (19)	8 (10.8)	NA	NA	88 (19.1)	113 (12.2)	1 (7.1)	10 (7.4)	6 (24.0)	54 (24.8)	16 (24)	NA	NA	NA	0.475	0.17–1.35	0.161
Prior CABG no. (%)	1 (4.8)	8 (10.8)	NA	NA	35 (7.6)	45 (4.9)	2 (14.3)	0	2 (8.0)	20 (9.2)	11 (16)	NA	NA	NA	0.351	0.06–2.04	0.244
Lung disease no. (%)	6 (28.6)	31(41.9)	NA	NA	14 (13.1)	79 (8.5)	2 (15.4)	24 (17.9)	7 (28)	52 (23.9)	NA	NA	21 (21.2)	14 (17.7)	0.87	0.64–1.18	0.369
Cerebrovascular disease no. (%)	3 (14.4)	9 (12.2)	NA	NA	13 (12.1)	108 (11.6)	0	10 (7.4)	4 (16)	27 (12.4)	NA	NA	NA	NA	0.923	0.60–1.42	0.718
Atrial fibrillation no. (%)	6(8.6)	24 (32.4)	NA	NA	16 (15.0)	171 (18.4)	0	11 (8.1)	6 (25.0)	54 (25.5)	9 (13.4)	NA	14 (14.1)	12 (15.2)	0.717	0.29–1.78	0.473
**Echocardio** **graphic findings**																	
Aortic valve mean gradient (mmHg)	51.0 (41.0–59.0)	49.5 (41.0–62.0)	NA	NA	50.5 ± 17.5	47.1 ± 16.4	48.1 ± 9.7	50.0 ± 16.0	46.0 ± 10.4	49.2 ± 16.8	NA	NA	60.63 ± 23.6	60.77 ± 22.6	0.14	−2.85–3.14	0.9251
Aortic valve area ± SD (cm^2^)	0.60 (0.50–0.80)	0.65 (0.55–0.80)	NA	NA	0.6 ± 0.2)	0.7 ± 0.2	0.7 ± 0.1	0.8 ± 0.2	0.67 ± 0.22	0.69 ± 0.23	NA	NA	0.53 ± 0.26	0.47 ± 0.33	0.05	−0.01–0.11	0.1291

*Values are mean ± SD, median (interquartile range), or n (%).*

* *Only rates of the whole population were available.*

† *Comparing characteristics of type 1 to type 0.*

† *Bold values refer to p <0.05 with significant difference between groups.*

*CI, Confidence Interval; CABG, coronary artery bypass graft; MD, Mean Difference; RR, risk ratio; NYHA, New York Heart Association; NA, not applicable; PCI, percutaneous coronary intervention; STS PROM, society of thoracic surgeons predicted risk of mortality*.

**Table 4:** Procedure characteristics of patients with bicuspid aortic stenosis who underwent transcatheter aortic valve replace in the included studies.

	**Jilaihawi**		**Xiong**	**Kumar**	**Yoon**		**Forrest**		**Ielasi**		**Hamdan**	**Ou**	**Guo**		***MD* or RR†**	**95% *CI***	***p***
	**Type 0**	**Type 1**	Overall*	Overall*	**Type 0**	**Type 1**	**Type 0**	**Type 1**	**Type 0**	**Type 1**	Overall*	Overall*	**Type 0**	**Type 1**			
Transfemoral access no. (%)	114 (87.7)	78 (97.5)	NA	NA	101 (94.4)	874 (94.3)	14 (100)	133 (98.5)	25 (100)	191 (88.5)	65 (97)	NA	NA	NA	0.952	(0.88–1.32)	0.30
Pre-dilation no. (%)	116/127 (91.3)	75 (93.7)	NA	NA	NA	NA	14 (100)	123 (90.4)	11 (44.0)	78 (35.8)	33 (49)	179 (98.9)	99 (100)	75 (94.9%)	**0927**	**0.0884**–**0.971**	**0.00147**
Post-dilation no. (%)	24/128 (18.8)	40 (50.0)	NA	NA	NA	NA	1 (7.1)	54 (40.0)	7 (28)	49 (22.5)	22 (33)	109 (60.2)	71 (71.7)	55 (69.6)	0.973	0.81–1.17	0.769
**Implanted valve type**																	
SEV no. (%)	60 (46.2)*		80 (100)	55 (82.1)	18 (16.8)	217 (23.4)	14 (100)	136 (100)	9 (36)	64 (29.4)	32 (48)	181 (100)	99 (100)	79 (100)	/	/	/
BEV no. (%)	70 (53.8)*		0	12 (17.9)	89 (83.2)	651 (70.2)	0	0	16 (64)	154 (70.6)	35 (52)	0	0	0	/	/	/

*Values are mean ± SD, median (interquartile range), or n (%).*

* *Only rates of the whole population were available.*

† *Comparing characteristics of type 1 to type 0.*

† *Bold values refer to p <0.05 with significant difference between groups.*

*CI, Confidence Interval; MD, Mean Difference; RR, risk ratio; NA, not applicable; SEV, self-expanded valve; BEV, balloon-expanded valve*.

### Post-TAVR PPI Between Sievers Type 1 and Type 0 Morphology

**Figure 2:**
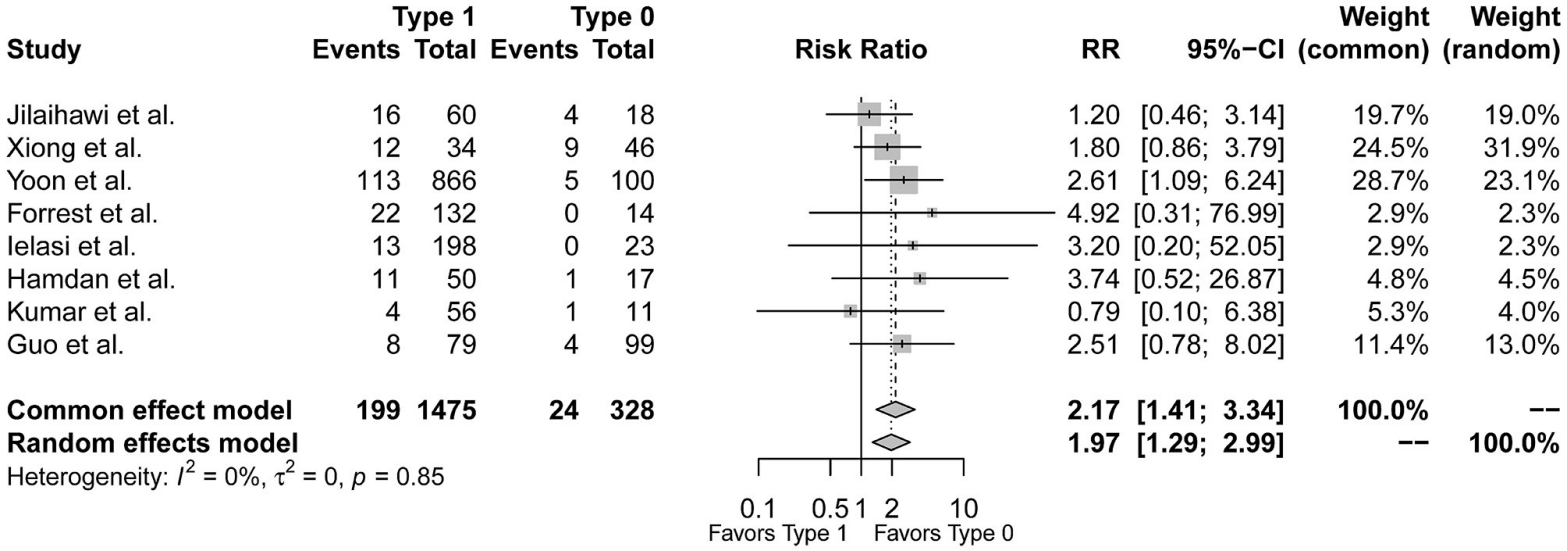
The forest plot of the pooled-analysis comparing post-TAVR permanent pacemaker implantation between Sievers type 1 and type 0 BAV morphology. RR, risk ratio; CI, confidence interval.

In the 9 included studies, the endpoint of the study of Ou et al. was solely post-TAVR HAVB rather than new PPI so included in the secondary analysis (10), Guo et al. and Hamdan et al. reported the incidence of composite conduction abnormalities besides new PPI (17, 22), and the other six only reported post-TAVR PPI rates. Therefore, the event rates of post-TAVR PPI between type 1 and type 0 BAV morphology were available in the 8 studies except for Ou et al. The pooled-analysis showed an increased risk of post-TAVR PPI for type 1 BAV morphology than type 0 (*RR* = 1.97, 95%*CI* 1.29–2.99, *p* = 0.0016) (Figure 2). After excluding the study of Yoon et al. as the only one with a significant difference between groups, the direction of pooled estimate was unchanged (*RR* = 1.81, 95%*CI* 1.12–2.91, *p* = 0.0152) which testified the stability of our pooled result (Supplementary Figure 1).

### Conduction Abnormalities Between Sievers Type 1 and Type 0 BAV Morphology

**Figure 3:**
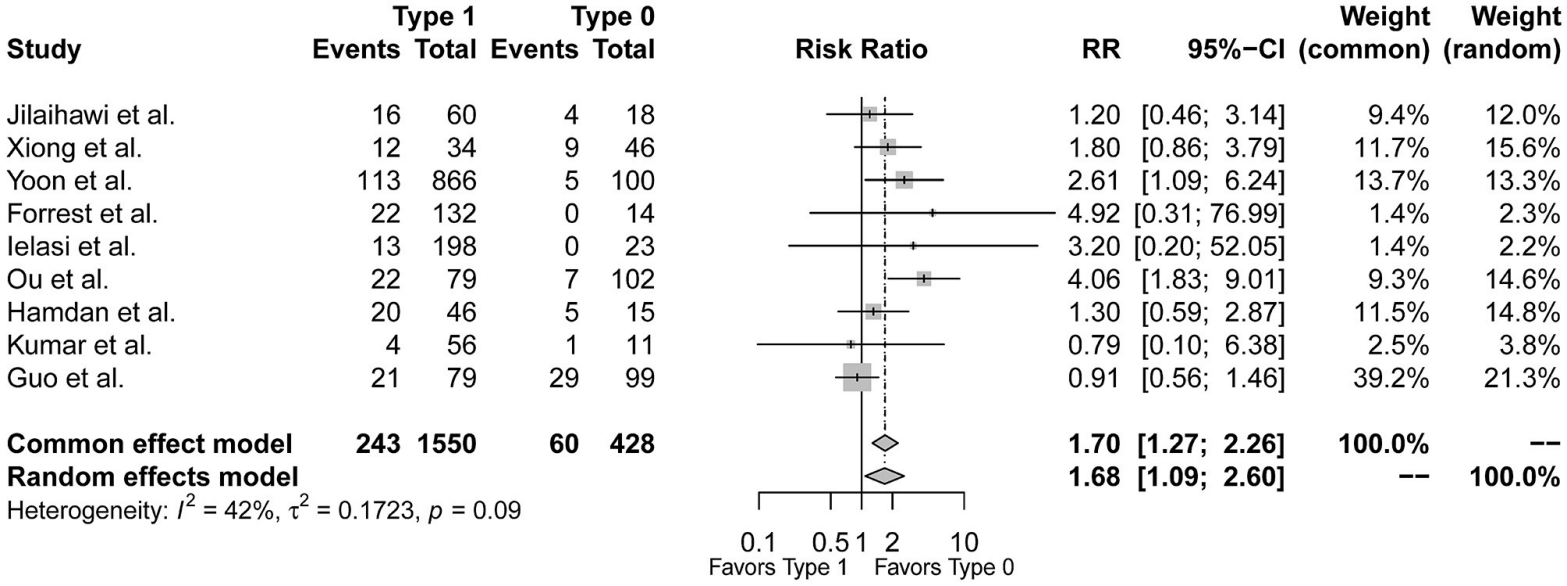
The forest plot of the pooled-analysis comparing post-TAVR conduction abnormalities between Sievers type 1 and type 0 BAV morphology. RR, risk ratio; CI, confidence interval.

**Figure 4:**
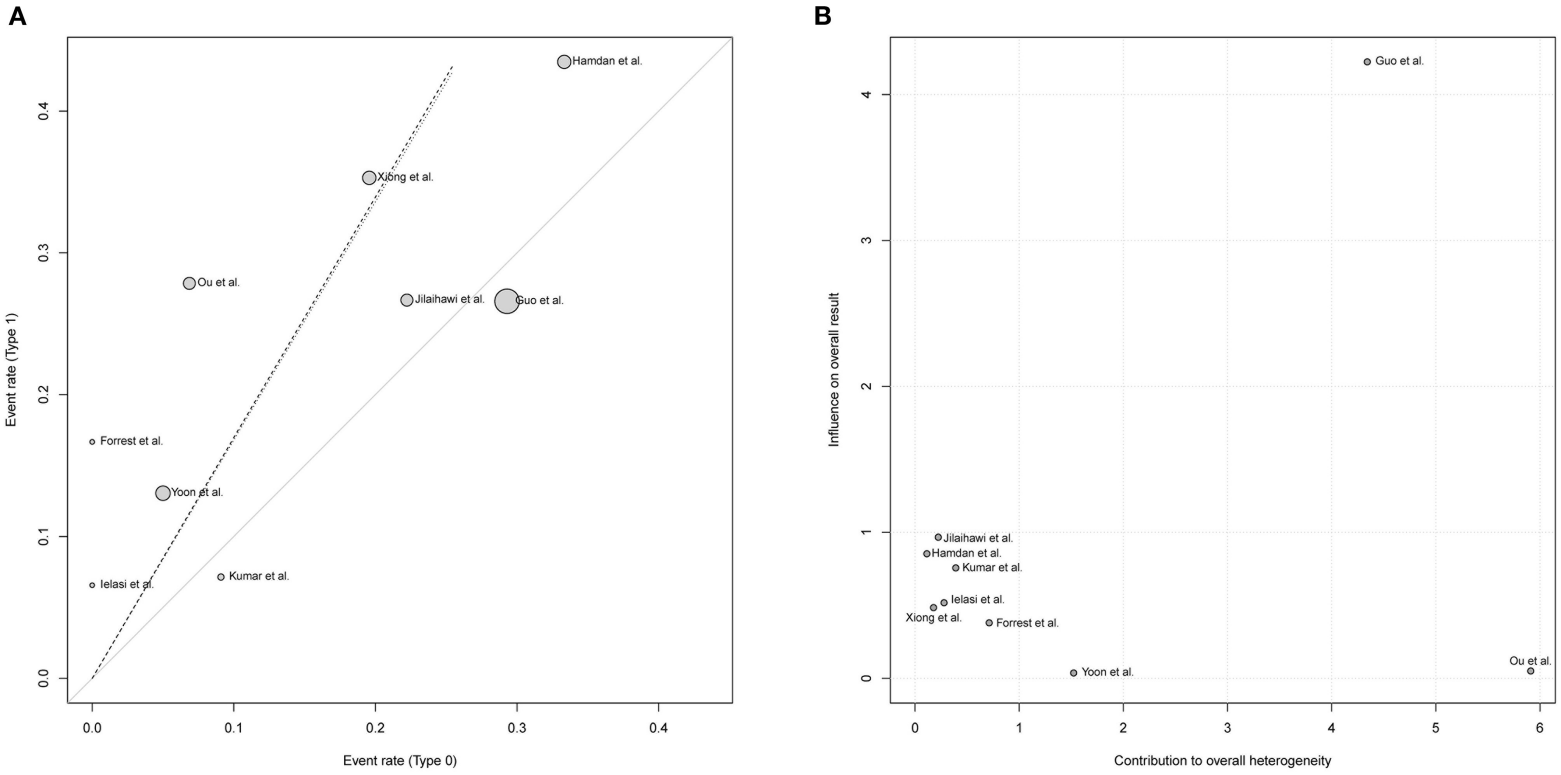
Exploring the heterogeneity in the pooled estimate of post-TAVR conduction abnormalities, L'Abbé plot **(A)** and Baujat plot **(B)** indicated the greatest contributor to heterogeneity was the study of Guo et al. followed by Ou et al. RR, risk ratio; CI, confidence interval.

Figure 3 demonstrated that the risk of post-procedural conduction abnormalities was significantly higher for type 1 BAV morphology compared to type 0 who underwent TAVR (*RR* = 1.68, 95%*CI* 1.09–2.60, *p* = 0.0195). The funnel plot was generally symmetric (Supplementary Figure 2). Notably, there was moderate heterogeneity in the pooled estimate (*I*^2^ = 42%, τ^2^ = 0.1723, *p* = 0.09). Drawing L'Abbé plot (Figure 4A) and Baujat plot (Figure 4B), we speculated the heterogeneity might be attributed most to the study of Guo et al., followed by Ou et al. Table 5 summarized the pooled estimate and corresponding heterogeneity by excluding the specific study/studies from the whole collection. The study of Yoon et al., as a secondary contributor to heterogeneity, was chosen as a comparator (Figure 4). After excluding Guo et al. and Ou et al., the heterogeneity dropped markedly to zero with direction of the estimate unchanged but additionally excluding Yoon et al. would not further decrease the heterogeneity.

**Table 5:** The pooled estimate and heterogeneity by excluding the specific study from the whole collection in the comparison of conduction abnormalities between type 1 and type 0 BAV morphology.

**The excluded study/studies**	***RR***	**95%** ***CI***		***p* for RR**	***I^**2**^*(%)**	***τ^2^***	***p* for heterogeneity**
		**Lower**	**Upper**				
Guo et al. and Ou et al.	1.68	1.13	2.50	0.0109	0	0	0.7857
Guo et al.	1.99	1.33	2.99	0.0009	0	0.0567	0.4338
Ou et al.	1.39	0.95	2.02	0.0906	0	0.0591	0.4360
Yoon et al.	1.57	0.98	2.53	0.0615	41.7	0.1830	0.1004

*BAV, bicuspid aortic valve; RR, risk ratio; CI, confidence interval*.

### Meta-Regression and Subgroup Analysis

The included observational studies would introduce huge confounding bias due to non-randomization. To adjust for confounding factors and further explore the heterogeneity, we performed random-effects univariate meta-regression and included mean difference of STS score, logarithmic RR of hypertension and diabetes which were statistically different between type 1 and type 0 at baseline in the regression. Age, sex, and New York Heart Association class as general effect modifiers were also included. Nevertheless, the analysis indicated no correlation between effect modifiers and the primary endpoint (Table 6), which might strengthen the reliability of our pooled estimate.

**Table 6:** Meta-regression analysis using potential confounding factors for post-TAVR PPI in the comparison of type 1 BAV to type 0.

**Covariates**	**Coefficient**	**95%** ***CI***		***p*-value**
		**Lower**	**Upper**	
MD of STS	−0.18	−1.56	1.2	0.799
MD of age	0.0728	−0.193	0.339	0.592
MD of Aortic area	1.1	−8.42	10.6	0.821
logRR of DM	1.63	−0.947	4.21	0.215
logRR of Male	−0.973	−5.89	3.94	0.698
logRR of NYHAIII-IV	2.01	−1.81	5.84	0.303
logRR of hypertension	0.678	−4.83	6.18	0.809
logRR of pre-dilation	−2.21	−21.4	17	0.822
NOS	−0.31	−1.1	0.49	0.450

*TAVR, Transcatheter aortic valve replacement; PPI, permanent pacemaker implantation; CI, Confidence interval; MD, Mean difference; logRR, logarithmic risk ratio; STS, Society of Thoracic Surgeons; DM, diabetes mellitus; NYHA, New York Heart Association; NOS, Newcastle-Ottawa Scale*.

In subgroup analysis limited to the SEV, the significance disappeared, and I^2^surged. In contrast, there is no heterogeneity detected by I^2^in the subgroup of SEV+BEV and the significance remained (Supplementary Figure 3). We failed to further stratify the analysis by valve generation due to most studies mixed with early- and newer- generations.

### Comparison Between Type 1 L-R and Non-L-R

**Figure 5:**
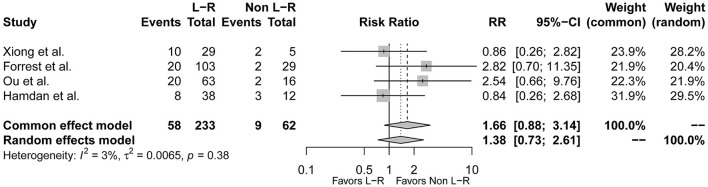
The forest plot of the pooled-analysis comparing post-TAVR conduction abnormalities between type 1 BAV morphology L-R and non-L-R subtype. RR, risk ratio; CI, confidence interval.

Type 1 L-R was reported to be related with more adverse events. To further explore the relationship between BAV morphology and conduction abnormalities, we compared the event rates between type 1 BAV subtypes. Of the included studies, 4 studies further reported incidence of post-TAVR conduction abnormalities in L-R, N-R, and N-L subtypes of type 1 BAV morphology (9, 10, 16, 17). However, the prevalence of L-N and R-N were relatively low in the included studies, so we combined L-N and R-N as a non-L-R group. Consequently, there were 233 patients with type 1 L-R subtype and 62 patients with non-L-R subtype. The pooled-analysis indicated that type 1 L-R was not associated with more post-TAVR conduction abnormalities at least compared to non-L-R (*RR* = 1.38, 95% *CI* 0.73–2.61, *p* = 0.32) (Figure 5).

## Discussion

This is the first pooled-analysis that focuses on the association of Sievers BAV morphology with post-TAVR conduction disorders. Our pooled-analysis demonstrated that there was a higher risk of post-TAVR PPI and conduction abnormalities for type 1 BAV morphology than for type 0.

The bicuspid aortic valve as the most common congenital heart disease affects 1~2% of the world population and is the predominant etiology for aortic stenosis in the young population (3). TAVR for tricuspid aortic stenosis has revealed its at-least non-inferiority to SAVR (1, 2) and are approved for BAV stenosis (7); however, previous studies demonstrated BAV stenosis who underwent TAVR suffered significantly higher risk of post procedural new PPI compared to SAVR (5–7). We failed to further reduce the incidence of post-TAVR PPI to the surgical benchmark for BAV stenosis even with the valve upgraded. Our analysis suggested Sievers BAV morphology might associate with post-TAVR PPI, which would facilitate accurately predicting conduction disorders.

Association of BAV morphology with conduction disorders was poorly discovered and such study was scarce. Current observations only indicated numerically but not a statistically higher incidence of post-TAVR PPI for type 1 BAV morphology than type 0. For example, in the Low Risk Bicuspid Study 22 in 132 type 1 needed PPI but none for type 0 (16). As for events other than PPI, Ou et al. proposed type 1 BAV morphology as an independent predictor of post-TAVR HAVB in the multivariable analysis, pitifully they failed to report the association with pacemakers (10). Kumar et al. reported 18 in 56 patients with type 1 developed new-onset LBBB after TAVR while the number of that for type 0 was 0 in 11 during 30-day follow-up (21). In the study of Shiyovich et al., BAV with raphe (type 1) compared to tricuspid counterparts had significantly increased risk of new-onset LBBB but BAV without raphe (type 0) did not, which supported the association of BAV morphology with conduction disorders in some degree (23).

Different from SAVR resecting the native valve, the valve in TAVR is reserved and has strong interaction with the implanted transcatheter heart valve (24). This could partially explain the association of BAV morphology with conduction abnormalities. Conduction abnormalities might result from injury of the conduction system, especially in the septum and the aortic root area during balloon expanding and valve implantation (8). In the view of mechanics, type 0 without fused raphe is in a relatively symmetric shape leading to less elliptical valve deployment and more symmetric distribution of contact pressure; however, raphe of type 1 might postpone symmetric expansion of implanted valve. Therefore, in the non-fused side of type 1 there was a smaller contact area with stent and resultant higher contact pressure than on the fused (25). Patient-specific simulation study indicated that there was higher contact pressure with the aortic root area in patients who experienced conduction disorders than in those who did not (26). Thus, type 1 BAV with raphe might enhance the contact pressure and increase the probability of conduction system injury.

In another view, there are several recognized risk factors for post-TAVR PPI in the BAV population. Deep implantation and oversizing of implanted valves would increase the chance to injure the conduction system (8–10, 17). Xiong et al. found that BAV patients complicated with post-TAVR PPI has significantly smaller sino-tubular junction diameter (9). Correspondingly Du et al. in their meta-analysis summarized type 1BAV morphology had smaller sino-tubular junction height and diameter than type 0. Therefore, we presumed valves implanted in type 1 might be relatively deeper and close to the membranous septum due to the smaller height and prone to be oversized due to the smaller diameter, which consequently damaged the conduction issue. For this reason, implantation depth and oversizing ratio as confounding factors should be adjusted but we failed to extract the data and include the factors in the meta-regression. Well-designed cohorts were warranted to further test our theory.

There was moderate heterogeneity in the pooled-analysis comparing the risk of post-TAVR conduction abnormalities between type 1 and 0. L'Abbé plot and Baujat plot indicated that the study of Ou et al. and Guo et al. contributed most to the heterogeneity, and the sensitivity analysis excluding the two showed I^2^ dropped sharply to zero, which verified their contribution to the heterogeneity. Actually, in the present pooled-analysis, conduction abnormalities were composite of HAVB, LBBB, and/or PPI. Endpoints in studies of Guo et al. and Hamdan et al. were truly composite, but that in the Ou et al. and the other studies were solely new-onset HAVB and post-TAVR PPI, respectively. Differences among the definitions may lead to heterogeneity. Accordingly, we performed the pooled-analysis with a concentration on post-TAVR PPI then set the composite conduction abnormalities as the secondary endpoint, and the pooled estimates of both suggested a higher risk for type 1 BAV morphology with consistency.

According to Sievers classification, type 1 could be further divided into 3 subtypes depending on the fused cups and raphe location, namely L-R, R-N, and L-N (14). Type 1 L-R was reported to be associated with more adverse events than type 1 N-R or N-L (10, 27, 28). Different from the anatomy of R-N and L-N, the non-fused side of type 1 L-R opposite to the fused raphe was near to the septum. Consequently, based on the mechanic theory mentioned above (25, 26), conduction issue enriched in the septum was prone to be hurt by increased contact pressure around the L-R non-fused side. L-R fusion as the most prevalent subtype in type 1 BAV morphology could be the driving factor that brought about the association of type 1 BAV morphology with conduction abnormalities and PPI. To test the hypothesis, we further compared the outcomes of L-R to non-L-R. Nevertheless, there was no difference between L-R and non-L-R in the risk of post-TAVR conduction abnormalities. Limited to a small subject number, we combined N-R and N-L up as non-L-R, which might decrease reliability of the evidence.

### Limitations

The pooled results should be interpreted with caution because of the following reasons. First, besides type 0 and type 1 BAV morphology, the Sievers classification also included type 2 and undetermined BAV morphology, but they were not included in our analysis because of their extremely low prevalence and scarce data. Second, all included studies were observational so adjustment for confounding factors was necessary. Meta-regression, we preformed, demonstrated no correlation between the effect modifiers and the pooled estimate, which relieved the confounding bias; however, there was relatively small number of studies included in the regression. Moreover, baseline conduction disorders, such as LBBB and RBBB, could be predisposing factors to post-TAVR HAVB and PPI, but such prevalence was seldom reported. Only Guo et al. presented a similar prevalence between type 1 and type 0, so the effect of baseline conduction disorders on the association was unknown. Last but not least, we must recognize the purpose of the pooled analysis was hypothesis-generating rather than proving type 1 BAV morphology as a strong predictor. We uncovered a rarely noticed association that needs more research to further validate.

## Conclusion

The current study found there was a higher risk of post-TAVR conduction abnormalities and PPI for Sievers type 1 BAV morphology than type 0, and the type 1 subtype L-R have no excess risk of post-TAVR conduction abnormalities compared to the non-L-R subtype. Our hypothesis that type 1 BAV morphology is a novel risk factor for conduction abnormalities warranted large cohorts to validate.

## Data Availability Statement

The original contributions presented in the study are included in the article/Supplementary Material, further inquiries can be directed to the corresponding authors.

## Author Contributions

JZ and XL independently completed the database searching, screening, and data extraction and wrote the manuscript. FX provided suggestions on statistical analysis and finished the pooled-analysis on R software. YC took responsibility for data checking and evaluated the eligibility of unsettled studies between JZ and XL. CL contributed to the discussion and revised the finished manuscript. All authors contributed toward data analysis, drafting and critically revising the paper, and agree to be accountable for all aspects of the work.

## Funding

This study was supported by the National Natural Science Foundation of China (82070388), Taishan Pandeng Scholar Program of Shandong Province (tspd20181220), and the National Natural Science Foundation of Shandong Province (ZR2020MH035).

## Conflict of Interest

The authors declare that the research was conducted in the absence of any commercial or financial relationships that could be construed as a potential conflict of interest.

## Publisher's Note

All claims expressed in this article are solely those of the authors and do not necessarily represent those of their affiliated organizations, or those of the publisher, the editors and the reviewers. Any product that may be evaluated in this article, or claim that may be made by its manufacturer, is not guaranteed or endorsed by the publisher.

## Abbreviations

TAVR: transcatheter aortic valve replacement
BAV: bicuspid aortic valve
PPI: permanent pacemaker implantation
HAVB: high-degree a trial-ventricle node block
LBBB: left-bundle branch block
SAVR: surgical aortic valve replacement
SEV: self-expanded valve
BEV: balloon-expanded valve
CI: confidence interval
RR: risk ratio.
