# Supplementary material for: Pooled-Analysis of Association of Sievers Bicuspid Aortic Valve Morphology With New Permanent Pacemaker and Conduction Abnormalities After Transcatheter Aortic Valve Replacement
Source: Front Cardiovasc Med. 2022 May 26;9:884911. doi: 10.3389/fcvm.2022.884911 (PMC9178076; doi:10.3389/fcvm.2022.884911)
Supplement: Supplementary file 1 [file Data_Sheet_1.PDF]

Supplementary Figure 1. Forest plot of the pooled -analysis comparing post-TAVR permanent pacemaker implantation between type1 and type 0 BAV morphology.

Study	Type 1 Events	Type 1 Total	Type 0 Events	Type 0 Total	RR	95%−CI	Weight (common)	Weight (random)
Jilaihawi et al.	16	60	4	18	1.20	[0.46; 3.14]	27.6%	24.7%
Xiong et al.	12	34	9	46	1.80	[0.86; 3.79]	34.3%	41.4%
Forrest et al.	22	132	0	14	4.92	[0.31; 76.99]	4.0%	3.0%
Ielasi et al.	13	198	0	23	3.20	[0.20; 52.05]	4.0%	2.9%
Hamdan et al.	11	50	1	17	3.74	[0.52; 26.87]	6.7%	5.9%
Kumar et al.	4	56	1	11	0.79	[0.10; 6.38]	7.5%	5.2%
Guo et al.	8	79	4	99	2.51	[0.78; 8.02]	15.9%	16.9%
**Common effect model**		**609**		**228**	**1.99**	**[1.22; 3.25]**	**100.0%**	**−−**
**Random effects model**					**1.81**	**[1.12; 2.91]**	**−−**	**100.0%**

Heterogeneity: $I^2 = 0\%$, $\tau^2 = 0$, $p = 0.83$

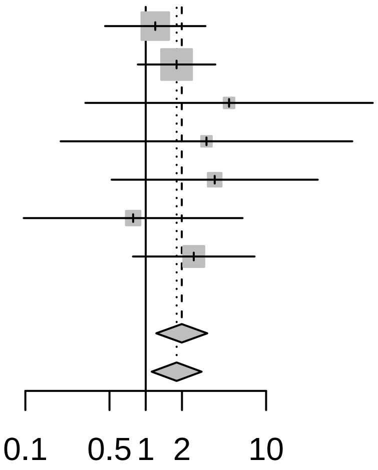

Supplementary Figure 2. Funnel plot of the pooled-analysis comparing post-TAVR conduction abnormalities between type1 and type 0 BAV morphology.

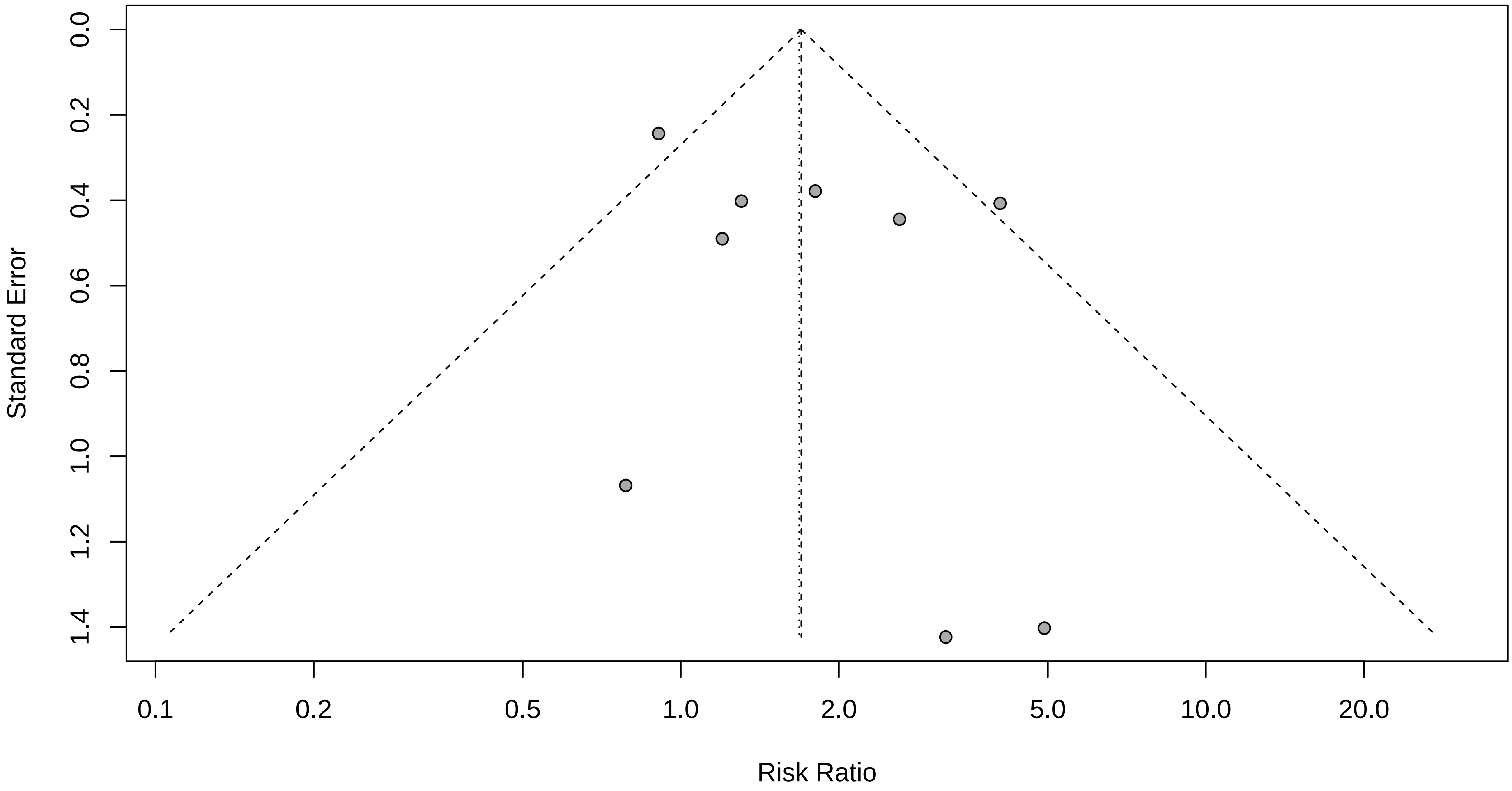

Supplementary Figure 3. Forest plot of the pooled -analysis comparing post-TAVR conduction abnormalities between type1 and type 0 BAV morphology stratified by valve type (SEV vs. SEV+BEV).

Study	Type 1 Events	Type 1 Total	Type 0 Events	Type 0 Total	RR	95%−CI	Weight (common)	Weight (random)
**valve = SEV+BEV**								
Jilaihawi et al.	16	60	4	18	1.20	[0.46; 3.14]	9.4%	12.0%
Yoon et al.	113	866	5	100	2.61	[1.09; 6.24]	13.7%	13.3%
Ielasi et al.	13	198	0	23	3.20	[0.20; 52.05]	1.4%	2.2%
Hamdan et al.	20	46	5	15	1.30	[0.59; 2.87]	11.5%	14.8%
Kumar et al.	4	56	1	11	0.79	[0.10; 6.38]	2.5%	3.8%
**Common effect model**		**1226**		**167**	**1.78**	**[1.08; 2.92]**	**38.4%**	**−−**
**Random effects model**					**1.57**	**[0.98; 2.54]**	**−−**	**46.1%**
Heterogeneity: $I^2 = 0\%$, $\tau^2 = 0$, $p = 0.65$								
**valve = SEV**								
Xiong et al.	12	34	9	46	1.80	[0.86; 3.79]	11.7%	15.6%
Forrest et al.	22	132	0	14	4.92	[0.31; 76.99]	1.4%	2.3%
Ou et al.	22	79	7	102	4.06	[1.83; 9.01]	9.3%	14.6%
Guo et al.	21	79	29	99	0.91	[0.56; 1.46]	39.2%	21.3%
**Common effect model**		**324**		**261**	**1.65**	**[1.16; 2.33]**	**61.6%**	**−−**
**Random effects model**					**1.92**	**[0.86; 4.29]**	**−−**	**53.9%**
Heterogeneity: $I^2 = 73\%$, $\tau^2 = 0.4230$, $p = 0.01$								
**Common effect model**		**1550**		**428**	**1.70**	**[1.27; 2.26]**	**100.0%**	**−−**
**Random effects model**					**1.68**	**[1.09; 2.60]**	**−−**	**100.0%**

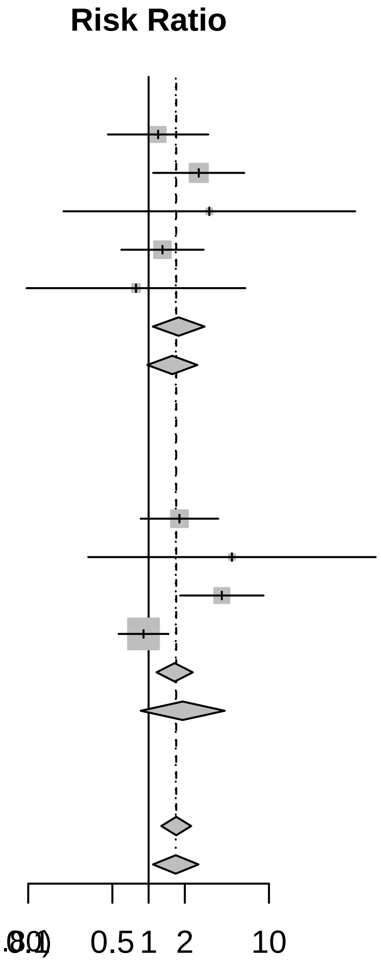

Heterogeneity: $I^2 = 42\%$, $\tau^2 = 0.1723$, $p = 0.09$

Test for subgroup differences (fixed effect): $\chi^2_1 = 0.06$, df = 1 ($p = 0.80$)

Test for subgroup differences (random effects): $\chi^2_1 = 0.17$, df = 1 ($p = 0.68$)
